# Supplementary material for: Patterns of Intron Gain and Loss in Fungi
Source: PLoS Biol. 2004 Nov 30;2(12):e422. doi: 10.1371/journal.pbio.0020422 (PMC532390; doi:10.1371/journal.pbio.0020422)
Supplement: Table S1 — Also available at http://genes.mit.edu/NielsenEtAl/. (4.3 MB ZIP). [file pbio.0020422.st001.zip › NielsenEtAl/html/755.html]

AN6272.1.NCU08005.1.MG01718.1.FG01927.1


```
 CLUSTAL W (1.82) Multiple Sequence Alignments - Introns Inserted


Sequence 1: AN6272.1	458 aa
Sequence 2: FG01927.1	522 aa
Sequence 3: NCU08005.1	444 aa
Sequence 4: MG01718.1	389 aa
Alignment Length: 546 aa
Number Identitical Residues: 131 aa
Alignment Score (without introns) 7134


MG01718.1 	--MFEALPVP--FGLVR------------------FGVAPDHP-----------------
NCU08005.1	-MTFVARVKPGALGLRGRWPSQVHAPRRMYSQHPAHHTSPEHPLRVAVIGSGPAGFYTTY
FG01927.1 	MVPSIVRRSGRLEALRYSFRLVSPQRQFSSSTARFSPRSPDAPFRMAVVGSGPAGFYTAY
AN6272.1  	------------------------------------------------------------
          	                                                            

MG01718.1 	----EVK0----------------~-------------~NCQDKFNEVAASPNFRFVGNV
NCU08005.1	KLMAKIQ~GTKVDMYESLPVPFGL0-------------~NCQEKFEEVASSPDFRFIGNV
FG01927.1 	RVMGKVP~GVKVDMYESLPVPFGL~VRHGVAPDHPEVK0NCQDKFDEIASQPNFSFVGNV
AN6272.1  	-------~-----MYEKLPVPFGL~ARYGVAPDHPEVK0NCEEKFTEVAASPRFNFIGNV
          	               .. . . .     . :.. .. . **::** *:*:.* * *:***

MG01718.1 	SIDRPSNHHGGATIPLSALLSNYHAVVFAYGAIQDRTLGILGEDQ-KGVYSAREFVGWYN
NCU08005.1	SVGTKSDHPDGLTVPLASLFRHYNAIIFAYGAAQDRKLGIPGEDQLKGVYSAREFVGWYN
FG01927.1 	SIGLPGHSSEHCTIELQNLMRHYDAVLFAYGASEDKKLGIPGESTLSNIHSAREVVGWYN
AN6272.1  	ELG--------ETLPLRVLKPHYDAILFAYGAPKDKKLGIQGEDALRSVYSAREFVGWYN
          	.:.         *: *  *  :*.*::***** :*:.*** **.   .::****.*****

MG01718.1 	GLPEHAGLDPDLTQGEEAVIIGQGNVALDVARMLLQDVDVLRKTDIAEHAVEALSRSRIK
NCU08005.1	GLPQHANLNPDLTQGEEAVVIGQGNVALDVARMLLEDVDVLRKTDIAAHALETLSQSRVK
FG01927.1 	GLPGCSGLNIDLTQAEEAVVIGQGNVALDVARMLLEDVDVLRKTDITEKALEALSRSRIK
AN6272.1  	GLPEHRDLNPDLAAGEDAVIIGQGNVALDVARILLSGVDRLRQTDIADYALETLSSSKIK
          	***   .*: **: .*:**:************:**..** **:***:  *:*:** *::*

MG01718.1 	RVHIVGRRGPMQ~AAFTIKELRELVKLDRVALHPVDASLVPNP--KEIKERPAKRLMDLL
NCU08005.1	RVHVVGRRGPMQ~AAFTIKEVRELMKLSNVSFHPVDTSLLPPD--LKSLPRAPRRLMEML
FG01927.1 	RVHVVGRRGPMQ0ASFTIKEVRELMNLKEVAFLPLNRSLIPEN--IKTLPRASKRLMEVL
AN6272.1  	RVRVVGRRGPLQ0ASFTIKEVRELLQLPSVSFDPIPRDLLPPEDVISALPRAQKRLIQLL
          	**::******:* *:*****:***::*  *:: *:  .*:*  .  .   *. :**:::*

MG01718.1 	LK---ASPVSPAESSKSWSLDFCLSPTQFISSPED----PSRLSATEFHRTIL-SNN-FD
NCU08005.1	AKGTTAISQSPSETAKSWSLDFCLTPKAFSPSSSSSTPTSTQLASTTFERTTL-SPSPFD
FG01927.1 	VK---GSPAPHDVVSKTWSLHSCLSPKNFLGNQND----PSKVASTEFDITEL--ASPFD
AN6272.1  	SK---GSTNDPTTTTKSWSLDFLLSPECLNWSPVF----PYRLSHVRFARNELDPADPFS
          	 *   . .      :*:***.  *:*  :  .       . ::: . *  . *.. ..*.

MG01718.1 	PKANVQ-----GISNETITMPSSVAFRSIGYKSVPLPSFSSLGIEFDEHRGILRNDGLGR
NCU08005.1	PNAYAL-----PTG-ETLTLPSSIAFRSIGYKSTPLPEFSDINIPFDERRGIISNDGRGR
FG01927.1 	PKSAVK----ATGKTALL--PSDVVFRSVGYKSIALPGFSEIGIQFDDRRGIVDNDGLGR
AN6272.1  	ASAKVRPKHLSDGKPAKVNIPANTFFRSVGYKGLPLPGLEDLGVPFDESRGVIPNDGFGR
          	..: . ..  :      :. *:.  ***:***. .** :..:.: **: **:: *** **

MG01718.1 	VARE0AGSRE---ALRASSHPLWRMLSLLVKPSWRTG~----IRVPSSSLLEMKKAPDPS
NCU08005.1	VQHE~ERTRG---AEMSHGS----FPGLYCAGWVKTG1----LGE-QDAIFECG--SERA
FG01927.1 	VTRM~VS------DAHAGGAHNEKVPGVYCAGWVKNG~PTGVIASTMQDAFTTGDAIVHD
AN6272.1  	VTVP~LASEAGTHDTLPNGSLISHLPGLYCAGWVKRG~PTGVIATTMTDAFSTADAIAAD
          	*      :  .:    . .     . .:      : * .:. :  .    :   .:    

MG01718.1 	SVGMG----------------------LPKKCR---------------------------
NCU08005.1	WVGRG----------------------EE-------------------------------
FG01927.1 	WLSGG-QFLNTSGNGNVTGWEGLRQDAGPTTCRAVEWDDWRKIDRAERERGQKIGKEREK
AN6272.1  	VAQHGNTLLNPSNERTGLGWEGVRAEAEQRGVRATTWEDWERIDRAERERGSLKSKPREK
          	    *.   ..:.. .  . ..   .:      :   ..    . :. . ..  ..  ..

MG01718.1 	------------
NCU08005.1	------------
FG01927.1 	FTRTEDMLAVLD
AN6272.1  	FGRVEEMLAAL-
          	    ..  :
```
